# Treatment of Steroid-Refractory Acute Graft-*Versus*-Host Disease Using Commercial Mesenchymal Stem Cell Products

**DOI:** 10.3389/fimmu.2021.724380

**Published:** 2021-08-19

**Authors:** Makoto Murata, Takanori Teshima

**Affiliations:** ^1^Department of Hematology and Oncology, Nagoya University Graduate School of Medicine, Nagoya, Japan; ^2^Department of Hematology, Hokkaido University Faculty of Medicine, Sapporo, Japan

**Keywords:** graft-*versus*-host disease (GVHD), mesenchymal stem cell (MSC), steroid, ruxolitinib, thymoglobulin

## Abstract

Acute graft-*versus*-host disease (GVHD) is a life-threatening complication that can develop after allogeneic hematopoietic stem cell transplantation. In particular, the prognosis of patients with steroid-refractory acute GVHD is extremely poor. Ryoncil™ (remestemcel-L), a human bone marrow-derived mesenchymal stem cell (MSC) product, failed to show superiority over placebo in patients with steroid-refractory acute GVHD, but it was approved for use in pediatric patients in Canada and New Zealand based on the results of a subgroup analysis. Temcell^®^, an equivalent manufactured MSC product to remestemcel-L, was approved in Japan based on small single-arm studies by using a regulation for regenerative medicine in 2016. The efficacy of Temcell was evaluated in 381 consecutive patients treated with Temcell during the initial 3 years after its approval. Interestingly, its real-world efficacy was found to be equivalent to that observed in a prospective study of remestemcel-L with strict eligibility criteria. In this article, the potential of MSC therapy in the treatment of acute GVHD is discussed. A meticulous comparison of studies of remestemcel-L and Temcell, remestemcel-L/Temcell and ruxolitinib, and remestemcel-L/Temcell and thymoglobulin showed that the precise position of remestemcel-L/Temcell therapy in the treatment of acute GVHD remains to be determined.

## Introduction

Acute graft-*versus*-host disease (GVHD) is a life-threatening complication that can develop after allogeneic hematopoietic stem cell transplantation (HSCT) (1, 2). Systemic corticosteroid is a standard first-line treatment, but the response rate ranges from 40% to 60% (3, 4). Many agents have been evaluated as second-line treatment for acute GVHD (5, 6). However, no consensus has been reached regarding the optimal approach for the management of steroid-refractory acute GVHD (SR-aGVHD) (7). A recent, randomized, phase 3 study comparing ruxolitinib and control (nine treatment options) for SR-aGVHD demonstrated a higher overall response (OR), defined as complete response (CR) and partial response (PR), in the ruxolitinib group (8). However, the study failed to demonstrate a significant advantage of using ruxolitinib in terms of overall survival (OS) or non-relapse mortality (NRM). Another recent, randomized, phase 3 trial comparing inolimomab and control (antithymocyte globulin) also demonstrated no significant advantage using inolimomab in terms of OS (9). Thus, no second-line treatment has been proven to improve survival in patients with SR-aGVHD.

Mesenchymal stem cells (MSCs) have been extensively studied as a treatment for SR-aGVHD (10). Efficacy of the commercial MSC product remestemcel-L (Ryoncil™, Mesoblast, Ltd, Melbourne, Australia; formerly Prochymal^®^, Osiris Therapeutics, Columbia, MD, USA) was evaluated in a randomized, phase 3 trial comparing the administration of remestemcel-L and of placebo in conjunction with another second-line therapy (11). Unfortunately, the study failed to meet its primary endpoint of durable CR and the secondary endpoint of the OR rate. Thus, despite a number of reports of positive outcomes of MSC therapy, unambiguous evidence of efficacy from randomized studies is still lacking (12).

However, the *post hoc* analyses of the randomized trial demonstrated that the pediatric patients, as well as the patients with liver involvement, in the remestemcel-L group had a significantly higher OR rate than those in the placebo group (11). In Japan, Temcell^®^ (JCR Pharmaceuticals Co. Ltd, Ashiya, Japan) was approved based on the results of small single-arm studies as “regenerative medicine” *via* a new Japanese initiative on stem cell therapies, which requires the results of additional clinical trials to confirm safety and to predict likely efficacy, in 2016. Temcell, which has no generic name, is the equivalent manufactured MSC product to remestemcel-L, derived from unrelated adult bone marrow. We recently reported the outcomes of 381 consecutive patients who were treated with Temcell during the initial 3 years after its approval (13). Interestingly, the treatment outcomes of Temcell in the real-world setting achieved an efficacy equivalent to that obtained in a prospective study of remestemcel-L. The multivariate analyses identified some factors to predict a higher OR rate and lower NRM after Temcell therapy in patients with SR-aGVHD.

This Mini Review article will discuss the potential of MSC therapy in the treatment of acute GVHD based mainly on data from the studies of a large number of patients receiving remestemcel-L or Temcell.

## A Brief Review of MSC Therapy for Acute GVHD

MSCs, which are alternatively defined as mesenchymal “stromal” cells, can be isolated and expanded from various tissues including bone marrow (14), umbilical cord (15), placenta (16), adipose tissue (17), and dental pulp (18). In the bone marrow, MSCs at different stages of maturation form the hematopoietic stem cell (HSC) niche, which play an important role in the maintenance and renewal of HSCs (19). These properties may contribute to facilitating the engraftment of transplanted HSCs, and therefore co-transplantation of MSCs with HSCs has been widely studied to promote engraftment in autologous and allogeneic HSCT (20–34). On the other hand, MSCs interact with the innate and adaptive immune systems *via* the direct cell-to-cell contact and the release of soluble factors, resulting in the regulation of immune activities (19). These properties may contribute to treating immune-mediated diseases, and therefore administration of MSCs has been widely studied to treat acute and chronic GVHD (11, 13, 35–64). A recent mouse study suggested that MSCs promoted the proliferation of innate lymphoid cells and their production of interleukin-22 (65), which stimulate proliferation and differentiation of intestinal stem cells to regenerate the damaged tissue (66). However, this remains to be proved clinically. Another recent study raised an alternative hypothesis that an apoptosis of infused MSCs by recipient cytotoxic cells may contribute to MSC-induced immunosuppression (67).

**Figure 1:**
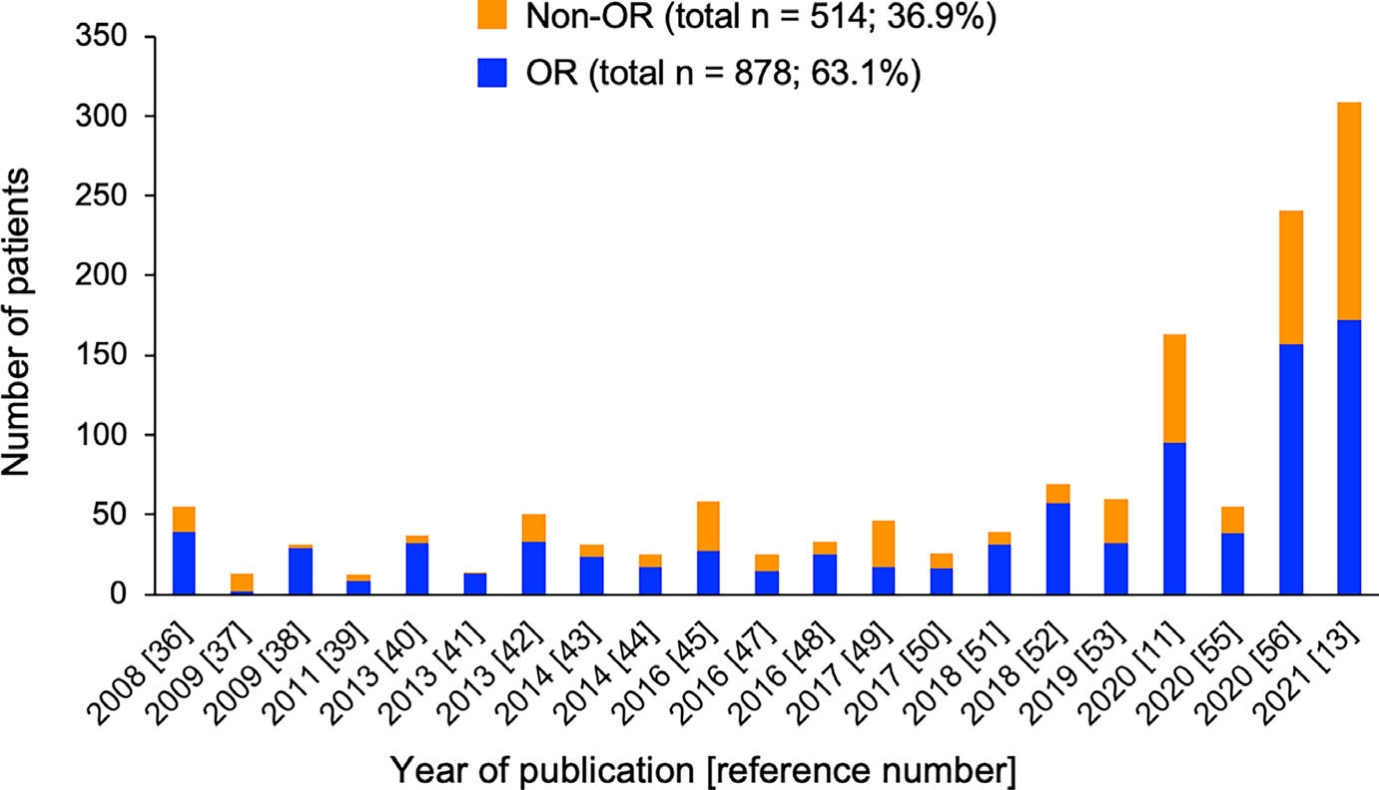
Number of patients with (blue) and without (orange) overall response (OR) in each study of MSC therapy for acute GVHD. Total numbers and percentages of patients with or without OR as the sum of all reported cases are provided.

MSCs were initially manufactured at each transplant hospital or factory by using various cell sources and culture methods, resulting in heterogeneity of MSCs (68). Thereafter, a commercial MSC product, remestemcel-L (Prochymal, currently Ryoncil), was developed in the United States (38). In Europe, a clinical-grade MSC product, called MSC-Frankfurt am Main, can now be used in clinical practice across several European countries (46, 52). According to the literature, more than 1400 patients have received MSCs as treatment of acute GVHD in the world (11, 13, 35–59). The numbers of acute GVHD patients treated with MSCs by year of publication are shown in **Figure 1**. Interestingly, there is a trend that OR rates were higher in previous studies than in recent larger studies. For all of these reported cases, the overall OR rate was 63%. Overall, MSC therapy is well tolerated. Infusion-related reactions were observed in 1.8% of patients who received remestemcel-L, less than that in patients who received placebo (2.5%) (11). No ectopic tissue formation has been reported. Although one retrospective study found a significant increase of pneumonia-related death with MSC therapy than without MSC therapy (59), another retrospective study demonstrated no difference in the risks of infections and relapse between MSC and non-MSC therapies (58). There were no differences in the rates of infection and relapse between the remestemcel-L and placebo groups in a randomized study (11).

In this review, we particularly focus on three major studies of commercial MSC products, including large number of patients (11, 13, 56), in order to avoid heterogeneity of MSC product.

## MSC Therapy With Remestemcel-L for Acute GVHD

A randomized, phase 3 trial of remestemcel-L *vs*. placebo added to another second-line therapy according to institutional standards in patients with SR-aGVHD was conducted (11). The main results of the study are summarized in **Table 1**. Of the 260 randomized patients, 163 received at least one infusion of remestemcel-L, and 81 received at least one infusion of placebo. Remestemcel-L therapy did not meet the primary endpoint of greater durable CR for at least 28 days within the first 100 days after enrollment (35% *vs*. 30%; *P* = 0.42). The proportions of patients achieving OR on day 28 (58% *vs*. 54%; *P* = 0.59) and OS on day 180 (34% *vs*. 42%; *P* = 0.60) were not different between the remestemcel-L and placebo groups.

**Table 1:** Comparison of outcomes among selected studies of therapy for acute graft-*versus*-host disease.

Report [reference number]	Kebriaei et al. (11)		Kurtzberg et al. (56)	Murata et al. (13)		Zeiser et al. (8)		Murata et al. (69)
Study design	Randomized, double-blinded		Single-arm, prospective	Retrospective		Randomized, open-label		Retrospective
Study therapy	Remestemcel-L+ another therapy	Placebo+ another therapy	Remestemcel-L	Temcell	Temcell	Ruxolitinib	Unspecified MSC	Thymoglobulin
Treatment(s) prior to study therapy	Steroid therapy only	Steroid therapy only	Multiple therapies	Multiple therapies	Steroid therapy only	Steroid therapy only	Steroid therapy only	Steroid therapy only
Number of patients	163	81	241	309	153	154	15	99
Median age (range), y	44 (0 to 70)	40 (0 to 70)	9 (0 to 18)	49 (0 to 72)	49 (0 to 70)	53 (12 to 73)	No data	39 (2 to 69)
Grade III-IV or C-D acute GVHD, %	77	74	80	80	79	64	No data	75
OR rate on day 28, %	58	54	65	56	61	62	60	60
NRM, %	No data	No data	No data	67 at 1y	59 at 1y	43 at 1y	No data	71 at 1y
OS, %	34 on day 180	42 on day 180	67 at day 100	27 at 1y	33 at 1y	49 at 1y	No data	27 at 1y
Infection								
Evaluation period	Within 180 days	Within 180 days	No data	Within 52 weeks	Within 52 weeks	Up to the data cut off*	No data	Within 100 days
At least one infection, %	88	82	No data	47	45	80	No data	59
Bacterial infection, %	No data	No data	No data	30	24	48	No data	28
Fungal infection, %	No data	No data	No data	9	6	17	No data	11
CMV antigenemia, %	No data	No data	No data	20	22	No data	No data	25
Viral infection, %	No data	No data	No data	42	40	57	No data	35

OR indicates overall response; NRM, non-relapse mortality; OS, overall survival; CMV, cytomegalovirus.

*The follow-up period ranged 0.03 to 23.62 months.

A single-arm expanded access treatment with remestemcel-L in 241 patients under the age of 18 years with acute GVHD resistant to multiple immunosuppressive therapies was conducted (56). The main results of the study are summarized in **Table 1**. Despite the fact that 50% of patients had grade D acute GVHD (70), and 79% were classified as high-risk acute GVHD (71), 65% of patients achieved OR on day 28. The achievement of OR on day 28 was associated with higher OS on day 100: 82% in patients with OR and 39% without OR (*P* < 0.0001, log-rank test).

## MSC Therapy With Temcell for Acute GVHD

More recently, real-world outcomes for 381 patients who received Temcell as a health insurance-covered treatment for acute GVHD were reported from Japan (13). The main results of the study are summarized in **Table 1**. Of the 309 patients, 56% achieved OR on day 28. Of the 153 patients who received Temcell as a second-line therapy following first-line steroid therapy for classic acute GVHD, 61% achieved OR on day 28. Thus, the treatment of acute GVHD with Temcell covered by health insurance in Japan achieved an efficacy equivalent to that obtained in prospective studies of remestemcel-L with strict eligibility criteria.

On multivariate analysis, liver involvement of acute GVHD and longer duration from first-line steroid therapy to second-line MSC therapy (≥14 days *vs*. <14 days) were associated with a lower OR rate. Older patient (18 to 49 years and ≥50 years *vs*. <18 years), higher grade of GVHD (III and IV *vs*. ≤II), higher number of GVHD therapies before MSC therapy (≥2 *vs*. ≤1), and non-achievement of OR on day 28 were associated with a higher NRM. OS was significantly higher in patients with an OR on day 28 than in those without an OR.

## Is the Efficacy of Remestemcel-L/Temcell Greater in Pediatric Patients?

A *post hoc* analysis of a randomized study demonstrated that the OR rate was significantly higher in the pediatric patients receiving remestemcel-L than in the pediatric patients receiving placebo (64% *vs*. 23%; *P* = 0.05) (11). However, it should be noted that only 13 pediatric patients were allocated to the placebo group, and only three (23%) of them achieved OR, in sharp contrast to the OR rate of 60% in 68 adult patients allocated to the placebo group (11). In the retrospective study of Temcell, there was no significant difference in OR rates among three age groups (< 18, 18 to 49, and ≥ 50 years) (13). Thus, there is not enough evidence to prove greater efficacy of remestemcel-L/Temcell in pediatric patients compared with adult patients.

## Is Remestemcel-L/Temcell Effective for Liver Acute GVHD?

A *post hoc* analysis of a randomized study demonstrated that the OR rate in patients with liver acute GVHD was significantly higher in the remestemcel-L group than in the placebo group (55% *vs*. 26%; *P* = 0.05) (11). OR rates in the remestemcel-L group were almost equal among the patients with liver, skin, and gut acute GVHD (55%, 58%, and 57%, respectively) (11). Similar results were obtained in a single-arm prospective study of remestemcel-L for pediatric patients; OR rates in the pediatric patients with liver, skin, and gut acute GVHD were 62%, 68%, and 65%, respectively (56).

On the other hand, a significantly lower OR rate in the patients with liver involvement of acute GVHD was reported in a retrospective study of Temcell; the OR rate in patients with liver acute GVHD was 36%, whereas OR rates in patients with skin or gut acute GVHD were 64% and 57%, respectively (13). Other immunosuppressants, such as ruxolitinib, antithymocyte globulin, and infliximab, are also reported to be less effective for liver acute GVHD than for skin or gut acute GVHD (69, 72–74). In the current situation where there is no fully effective treatment for liver acute GVHD, remestemcel-L/Temcell is an option for liver acute GVHD, but the efficacy of MSC therapy for liver acute GVHD remains unclear.

## When is the Best Time to Initiate Remestemcel-L/Temcell Therapy?

In a retrospective study of Temcell, multivariate analysis of all evaluable patients demonstrated a lower NRM in patients with no or one GVHD therapy before Temcell therapy compared with two or more GVHD therapies (odds ratio, 0.65; 95% confidence interval, 0.47 to 0.91) (13). Multivariate analysis of patients who received second-line Temcell therapy following first-line steroid therapy demonstrated a higher OR rate in patients with < 14 days than ≥ 14 days between first-line steroid therapy and second-line Temcell therapy (odds ratio, 2.27; 95% confidence interval, 1.09 to 4.76) (13).

In contrast, a single-arm prospective study of remestemcel-L for pediatric patients demonstrated no significant difference in the OR rate among the three groups of durations between first-line therapy and remestemcel-L therapy (74% for ≤ 14 days, 56% for 15 to 28 days, and 67% for ≥ 28 days). Thus, although it is possible that early initiation of remestemcel-L/Temcell therapy may have an advantage in terms of a higher response rate and/or lower NRM, further analysis of a larger cohort is required to provide an accurate answer to this question.

## How Many Infusions of Remestemcel-L/Temcell Are Required to Obtain a Response?

Remestemcel-L/Temcell at a dose of 2 × 10^6^ cells/kg was infused twice weekly for 4 consecutive weeks with additional infusion once weekly for a further 4 weeks in prospective studies (11, 41, 47, 55, 56). Analysis of real-world data of Temcell in Japan demonstrated that 61% of patients received ≤ 8 infusions, and 39% received 9 to 12 infusions (13). In patients who achieved OR by Temcell therapy, 50% and 90% achieved it by day 15 and day 28, respectively. These data suggest that less than 8 infusions may be sufficient in most patients, whereas more than 8 infusions are required only in a small population of patients.

On the other hand, 10% of the patients who achieved OR by day 28 with 8 infusions of Temcell experienced GVHD relapse as of day 90. Taken together, the administration schedule of remestemcel-L/Temcell therapy has not been optimized, or it has to be individually optimized.

## Which Is More Effective for Acute GVHD, Ruxolitinib or Remestemcel-L/Temcell?

It must be stated again that ruxolitinib is the only drug that has been proven to be significantly more effective than control (nine therapies) in a randomized, open-label trial (8). Ruxolitinib inhibits Janus kinase 1/2 signaling, resulting in the blockade of multiple cytokines, dendritic cell activation, and neutrophil activation (75). The main results of this study (8) are summarized in **Table 1**. The patients assigned to the ruxolitinib therapy (n = 154) achieved an OR rate of 62% on day 28, which was significantly higher than that (39%) in 155 patients receiving control therapy (*P* < 0.001). MSC was one of nine options for patients assigned to the control therapy; 15 patients received MSCs and 9 (60%) of them achieved OR on day 28 (**Table 1**). Although this study was not designed to compare ruxolitinib and each control treatment, OR rates in the patients receiving ruxolitinib and MSCs were similar (8). This has been the only prospective comparison of efficacy between ruxolitinib and MSC therapies for treatment of SR-aGVHD.

Unfortunately, NRM in the patients receiving MSCs as a control therapy was not analyzed in the randomized trial (8). Although NRM cannot be directly compared between prospective and retrospective studies, NRM at 1 year in patients with ruxolitinib therapy in a prospective study (8) was much lower than that in patients with Temcell therapy in a retrospective study (13) (43% *vs*. 59%). Future comparative studies between ruxolitinib and MSC therapies, in which the primary endpoint is defined as NRM or OS, but not the response rate, are of interest.

The most common adverse events of ruxolitinib therapy have been reported to be thrombocytopenia, anemia, and cytomegalovirus infection (8). The incidences of each infection after Temcell therapy (13) might be lower than those after ruxolitinib therapy (8) (**Table 1**). However, due to a difference in the evaluation period and a lack of detailed information about clinical course, it is not possible to conclude that Temcell therapy is less likely to cause infection compared with ruxolitinib therapy.

## Which Is More Effective for Acute GVHD, Antithymocyte Globulin or Remestemcel-L/Temcell?

Antithymocyte globulin affects not only T cells, but also B cells, dendritic cells, regulatory T cells, and natural killer T cells, resulting in its diverse effects on the immune system (76). The major adverse events of antithymocyte globulin therapy are infusion reaction and viral and fungal infections (77). There has been no prospective study comparing antithymocyte globulin and MSC therapies for acute GVHD treatment. However, the outcome of 99 patients who received thymoglobulin as a second-line treatment for SR-aGVHD (69) was comparable to that of 153 patients who received Temcell as a second-line treatment for SR-aGVHD (13). Both retrospective studies included consecutive patients during the initial three or four years after their health insurance approval in Japan.

The main results of the thymoglobulin study are summarized in **Table 1**. The OR rate on day 28 in the thymoglobulin study (60%) was equal to that of Temcell therapy (61%). However, NRM at 1 year was higher with thymoglobulin therapy than with Temcell therapy (71% *vs*. 59%). This difference resulted from neither patient age nor severity of GVHD, because the median age was not higher, but rather lower in the thymoglobulin study than in the Temcell study (39 *vs*. 49 years, respectively), and the proportion of grade III to IV acute GVHD was slightly lower in the thymoglobulin study than in the Temcell study (75% *vs*. 79%, respectively). Of note, the incidence of any additional infection within the first 100 days after the start of thymoglobulin therapy was 59%, whereas that within 52 weeks after Temcell therapy was 45% (**Table 1**), suggesting that Temcell therapy may have an advantage of a lower NRM associated with infectious complications compared with thymoglobulin therapy. Further analysis with detailed information, such as severity and therapeutic response of each infection, is required.

## Has There Been a Study to Compare MSCs and Other Therapies for Acute GVHD?

There have been no other prospective or retrospective studies to compare the efficacy of MSCs and other immunosuppressants in the treatment of acute GVHD.

## Conclusion

It is known that the incidence of severe acute GVHD is lower in Japanese than Caucasian patients (78), but the outcome of SR-aGVHD seems to be equally poor (4). Thus, effective second-line treatments for SR-aGVHD are an unmet need. Ruxolitinib is widely used as an acute GVHD treatment in the United States and Europe, but the use of MSCs has not been approved as a health insurance treatment in those countries. In contrast, Temcell is widely used in Japan, but ruxolitinib remains under review. Thus, it is currently impossible to compare the efficacy of remestemcel-L/Temcell and ruxolitinib in a real-world setting. As described in an earlier section, there has been a randomized, prospective study of remestemcel-L and placebo (11). The correct interpretation of the study is that the addition of remestemcel-L to another second-line therapy was not superior to a second-line therapy without remestemcel-L. In other words, the efficacy of remestemcel-L alone has not been prospectively compared with other immunosuppressive drugs in the treatment of acute GVHD.

In conclusion, the appropriate use of remestemcel-L/Temcell for acute GVHD remains to be determined. Future study is needed to establish more precisely the position of remestemcel-L/Temcell in the treatment of acute GVHD.

## Author Contributions

MM designed the review and wrote the manuscript, and TT wrote the manuscript and supervised the process. All authors contributed to the article and approved the submitted version.

## Funding

This work was supported in part by a Practical Research Project for Allergic Diseases and Immunology (Research on Technology of Medical Transplantation) (JP21ek0510032 to MM) from the Japan Agency for Medical Research and Development (AMED) and by a Grant-in-Aid for Scientific Research (21K08387 to MM) from the Japan Society for the Promotion of Science (JSPS).

## Conflict of Interest

MM has received honoraria from Kyowa Kirin, Sumitomo Dainippon Pharma, FUJIFILM, Toyama Chemical, Novartis Pharma, MSD, JCR Pharmaceuticals, Astellas Pharma, Miyarisan Pharmaceutical, Asahi Kasei Pharma, GlaxoSmithKline, Celgene and Otsuka Pharmaceutical. TT has received grants from Kyowa Kirin, Chugai, Sanofi, Astellas, TEIJIN PHARMA, Fuji Pharma and NIPPON SHINYAKU; personal fees from Novartis, Merck, Kyowa Kirin, Takeda, Pfizer and Bristol-Myers Squibb; and non-financial support from Janssen and Novartis.

## Publisher’s Note

All claims expressed in this article are solely those of the authors and do not necessarily represent those of their affiliated organizations, or those of the publisher, the editors and the reviewers. Any product that may be evaluated in this article, or claim that may be made by its manufacturer, is not guaranteed or endorsed by the publisher.
